# Combined Surgical Approach for Obstructive Sleep Apnea Patient

**DOI:** 10.1155/2018/4798024

**Published:** 2018-03-28

**Authors:** Mahmoud A. K. Ebrahim, Osamah AlSanea, Abdulmohsen E. Al-Terki

**Affiliations:** ^1^Al-Sabah Hospital, Ministry of Health, Kuwait City, Kuwait; ^2^Department of Surgery Health, Medical Clinic Dammam, Saudi Arabia; ^3^ENT Clinic, Dasman Diabetes Institute, Kuwait City, Kuwait

## Abstract

Obstructive sleep apnea (OSA) is a disease that is associated with high morbidity and mortality and can significantly impact the quality of life in a patient. OSA is strongly associated with obesity, and literature showed that weight loss will lead to improvement in OSA. The gold standard treatment for OSA is continuous positive airway pressure (CPAP). However, other methods of treatment are available. One of these methods is multilevel sleep surgery (MLS). Literature showed that bariatric surgery can also improve OSA. A common question is which surgical procedure of these two should be performed first. We present a 5-year follow-up of a patient who underwent simultaneously bariatric surgery and MLS. His apnea-hypopnea index (AHI) decreased from 53 episodes per hour to 5.2 per hour within the first 18 months, which was measured via a level 3 polysomnography. Five years after the surgery, a repeat level 3 polysomnography showed an AHI of 6.8 episodes per hour, and the patient is asymptomatic. The patient maintained his weight and did not use CPAP after the combined surgery during the five-year period.

## 1. Introduction

Obstructive sleep apnea (OSA) is characterized by recurrent upper airway obstruction during sleep and presents as oxygen desaturation, hypercapnia, and sleep fragmentation, which occurs because of complete or partial upper airway obstruction [1]. The OSA prevalence is approximately 2%–4% in middle-aged adults [2]. OSA is related to major metabolic, cardiovascular, and neurocognitive morbidities because of hypoxemia, hypercapnia, and sleep disturbance [1]. Continuous positive airway pressure (CPAP) is the current treatment of choice for moderate-to-severe OSA [3]. In compliant patients, CPAP improves sleepiness and quality of life and reduces cardiovascular morbidity [4]. Surgery is a second-line treatment for patients who cannot tolerate CPAP or CPAP nonresponders [5]. Because OSA involves one or more upper airway levels, a multilevel single-stage procedure has been developed to treat OSA (success rate of 60%) [6]. We present the 5-year follow-up data of a patient who underwent combined multilevel surgery (MLS) and laparoscopic Roux-en-Y gastric bypass. To our knowledge, this is the first such case in the literature. The patient exhibited significantly reduced apnea-hypopnea index (AHI) values without CPAP use.

## 2. Case Presentation

A morbidly obese male patient (body mass index (BMI) of 39 kg/m^2^) in his thirties, with a history of diabetes, hypertension, OSA, hypercholesterolemia, and hyperuricemia, consulted for excess body weight and sleep apnea; he had undergone many failed trials, including diets, exercise, and medication, to lose weight. He had experienced multiple episodes of apnea and gasping for air during sleep, snoring, and sleeping during the morning with headaches. He was put on CPAP but could not tolerate it and opted for an alternative treatment. Ear, nose, and throat examination revealed hypertrophied tonsils and inferior turbinates, with a redundant soft palate. His blood investigations were within normal range. Computed tomography of the paranasal sinuses revealed normal findings. His Epworth sleepiness scale (ESS) score was 18.

Preoperative level III polysomnography detected AHI of 53 episodes/h, indicating severe OSA. Sleep endoscopy showed that the tongue base did not fill the vallecula, but the lateral pharyngeal wall was collapsed where the palatine tonsils blocked the airway, plus at the level of the soft palate it showed a flaccid soft palate.

In June 2012, after easy intubation, the patient underwent MLS, which involved the insertion of five pillar palatal implants in the soft palate, followed by cold dissection tonsillectomy. Because the posterior end of the right inferior turbinate was blocking the posterior choanae, it was removed using zero-degree nasal endoscopy and a straight shaver. Bilateral blow-out fractures of the right and left inferior turbinates were performed to make the posterior nasal cavity patent. Once homeostasis was achieved, laparoscopic Roux-en-Y gastric bypass was immediately conducted. Postoperatively, the patient was admitted for 48 h intensive care unit monitoring and used CPAP for several days; he was discharged 4 days postoperatively.

At 18 months postoperatively, repeat level III polysomnography showed that AHI decreased from 53 to 5.2 episodes/h without CPAP use, and the patient was asymptomatic.

Five years later, follow-up level III polysomnography revealed AHI of 6.8 episodes/h, and the patient remains asymptomatic (current ESS score of 8 (versus 18 preoperatively); latest BMI, 27 kg/m^2^).

## 3. Discussion

Most OSA patients have multilevel disease; hence, nasal, oropharyngeal and palatal, and hypopharyngeal treatment measures are required to improve the airway [7]. Waite et al. first presented a multilevel procedure in 1989 [8]. Staged surgery with multilevel treatment can be used to treat OSA [9]. The aim of successful surgery is to reduce AHI to <20 or by ≥50% of preoperative AHI, which occurred in our patient [10]. Additionally, improvement in daytime sleepiness and reduction in ESS score to <10 (reduced to 8 in our case) are expected [11]. Until 2010, 11 controlled studies and 32 case series investigated the effectiveness of MLS. Average AHI was reduced to 20.3 postoperatively from 43.9 preoperatively. However, it remains unclear which combination of MLS is superior and most effective [12]. A meta-analysis of 1978 patients showed that the overall success rate was 66.4% in patients with multilevel sleep apnea surgery involving at least two anatomical sites: nose, oropharynx, or hypopharynx [13]. Furthermore, patients receiving postoperative CPAP had lower AHI and ESS scores and higher SpO_2_ [14]. Azbay et al. reported that approximately 48% of patients who cannot tolerate CPAP preoperatively can tolerate it postoperatively [14].

Two most important factors that determine postoperative AHI after multilevel surgery are preoperative AHI and tonsillectomy. Preoperative BMI is marginally significant, whereas age is not [15]. Tonsillectomy is proven to treat OSA [16]. MLS with tonsillectomy was effective in reducing AHI scores in 58% patients (versus 19% patients without tonsillectomy) similar to what has been previously reported [17]. Another important factor for maintaining the success rate of MLS postoperatively is sustained weight loss [18]. Neruntarat showed that, in most patients, MLS treatment failure was due to weight gain [19]. In our case, BMI was maintained even at 5 years postoperatively. Pillar implants are designed to reduce vibration or narrowing of the soft palate by increasing its stiffness and to decrease collapsibility of the airway [20–22] and have been reported to significantly decrease ESS and AHI [23, 24]; however, this finding is not definitive because only two of the studies reporting this result were placebo controlled [23, 24].

Obesity and OSA are known to be related [25]. Bariatric surgery can reduce many comorbidities, such as OSA, in obese people [26]. OSA prevalence in bariatric surgery patients is 64%–97%, with a strong male dominance [27]. Dixon et al. demonstrated that shorter sleep duration is associated with significant weight gain and high BMI, which can be attributed to reduced sleep efficiency and changes in sleep architecture [28]. Almost all studies in a meta-analysis reported reduction in AHI after bariatric surgery [29], but some studies stated that a longer follow-up period is associated with a larger relapse rate [30]. Conversely, a recent study showed that reduction in AHI can be maintained in the long term [31]. A meta-analysis by Buchwald et al. demonstrated that OSA resolved in 85.7%, patients assessed either subjectively or objectively (via postoperative polysomnography) [32]. Conversely, Greenburg et al. assessed OSA patients' AHI via polysomnography before and after bariatric surgery and detected significant residual OSA (AHI >15 events/h) in approximately 62% patients [33]. Holty et al. reported a huge reduction in ESS scores at 2 years after bariatric surgery in OSA patients [34]. Additionally, patients who underwent gastric bypass had greater improvement in AHI than patients who underwent laparoscopic adjustable gastric banding [35]. This can be explained by weight-dependent factors that decrease the mechanical force on the cervical region, upper airway, and diaphragm, as well as weight-independent metabolic effects such as bile flow alteration, restriction of gastric size, anatomical gut rearrangement and altered flow of nutrients, vagal manipulation, and enteric gut hormone modulation [35].

## 4. Conclusion

OSA affects multiple organ systems and can lead to significant morbidity and mortality. Although CPAP is the gold standard, several other methods, including surgery, have been developed to treat OSA. MLS is an alternative method for treating OSA, improving AHI, alleviating symptoms, and improving quality of life. This is a case report of a patient who underwent bariatric and sleep surgery simultaneously. Five-year follow-up revealed significant improvement and sustainability of the reduction in AHI and symptoms.
